# The effect of parameter variability in the allometric projection of leaf growth rates for eelgrass (*Zostera marina* L.) II: the importance of data quality control procedures in bias reduction

**DOI:** 10.1186/s12976-015-0025-y

**Published:** 2015-12-01

**Authors:** Héctor Echavarría-Heras, Cecilia Leal-Ramírez, Enrique Villa-Diharce, Nohe R. Cazarez-Castro

**Affiliations:** Centro de Investigación Científica y de Estudios Superiores de Ensenada, Carretera Ensenada-Tijuana No. 3918, Zona Playitas, Código Postal 22860, Apdo. Postal 360 Ensenada, B.C. Mexico; Centro de Investigación en Matemáticas, A.C. Jalisco s/n, Mineral Valenciana, Guanajuato Gto., Código Postal 36240 Mexico; Instituto Tecnológico de Tijuana, Calzada Tecnológico S/N, Fracc. Tomas Aquino, Tijuana, Baja California Código Postal 22414 Mexico

**Keywords:** Eelgrass leaf growth rates, Allometric estimation, Parametric uncertainty effects, Data quality control

## Abstract

**Background:**

Eelgrass grants important ecological benefits including a nursery for waterfowl and fish species, shoreline stabilization, nutrient recycling and carbon sequestration. Upon the exacerbation of deleterious anthropogenic influences, re-establishment of eelgrass beds has mainly depended on transplantation. Productivity estimations provide valuable information for the appraisal of the restoration of ecological functions of natural populations. Assessments over early stages of transplants should preferably be nondestructive. Allometric scaling of eelgrass leaf biomass in terms of matching length provides a proxy that reduces leaf biomass and productivity estimations to simple measurements of leaf length and its elongation over a period. We examine how parameter variability impacts the accuracy of the considered proxy and the extent on what data quality and sample size influence the uncertainties of the involved allometric parameters.

**Methods:**

We adapted a Median Absolute Deviation data quality control procedure to remove inconsistencies in the crude data. For evaluating the effect of parametric uncertainty we performed both a formal exploration and an analysis of the sensitivity of the allometric projection method to parameter changes. We used parameter estimates obtained by means of nonlinear regression from crude as well as processed data.

**Results:**

We obtained reference leaf growth rates by allometric projection using parameter estimates produced by the crude data, and then considered changes in fitted parameters bounded by the modulus of the vector of the linked standard errors, we found absolute deviations up to 10 % of reference values. After data quality control, the equivalent maximum deviation was under 7 % of corresponding reference rates. Therefore, the addressed allometric method is robust. Even the smaller sized samples in the quality controlled dataset produced better accuracy levels than the whole set of crude data.

**Conclusions:**

We propose quality control of data as a highly recommended step in the overall procedure that leads to reliable allometric surrogates of eelgrass leaf growth rates. The proliferation of inconsistent replicates in the crude data points towards the importance of discarding incomplete leaves. We also recommend avoiding errors in estimating the biomass of small leaves for which precision of the used analytical scale might be an issue.

## Background

Eelgrass is a relevant seagrass species that distributes worldwide in estuaries and nearshore environments. Eelgrass meadows provide habitat and foraging grounds for marine animals, buffers the shoreline from erosion, filter the water, and oxygenate the sediments. Recently at a global scale, deleterious effects derived from anthropogenic influences, have been exacerbated to such an extent that in the absence of remediation efforts, the important ecological services resulting from the permanence of eelgrass meadows could be irreversibly lost. Due to its ecological relevance eelgrass has been the subject of intense research and conservation efforts that are mainly carried out by means of transplanting plots. The measurement of biomass and productivity, provide key information for the evaluation of the overall status of a given eelgrass population. The noticeable growth form of this species makes the average rate of leaf growth per shoot-day measured over a growing interval of length ∆*t* determinant of overall productivity. In what follows we denote the biomass of an individual eelgrass leaf at time *t* through the symbol *w*(*t*) and its corresponding length by means of *l*(*t*). Similarly, we will symbolize the observed values of the biomass of leaves in shoots by means of *w*_*s*_(*t*) and the average rate of leaf growth per shoot-day through *L*_*g*_(*t*, ∆*t*). Conventional techniques for the assessment of *L*_*g*_(*t*, ∆*t*), require intensive sampling that involves the removal of shoots, and then tedious, time consuming dry weight measurement procedures in the laboratory. Even though the elimination of eelgrass shoots linked to a typical evaluation activity does not infringe damage to natural populations, the effects of shoot removal could be severe for transplanted plots. Therefore, in an overall program that leads to eelgrass conservation it is important to include nondestructive assessment methods [1, 2].

Bivariate allometric scaling relationships between measured quantities *X* and *Y* expressed as a power function of the form *Y* = *βX*^*α*^, appear in a myriad of research problems in physics, biology, and earth and planetary sciences [3–11]. The parameter α is named allometric exponent and *β* termed normalization constant. Particularly, Echavarria-Heras et al. [1] and Echavarria-Heras et al. [2], stressed the significance of the model

1 $$ w(t)=\beta l{(t)}^{\alpha }, $$

in the adaptation of an allometric proxy for *L*_*g*_(*t*, ∆*t*). This allometric surrogate will be here denoted by means of the symbol *L*_*ga*_(*α*, *β*, *t*, ∆*t*), and its explicit formulae derived in Appendix A.

The appropriateness of this device to provide truly nondestructive assessments depends on the time invariance of the parameters *α* and *β*, because estimates previously fitted at a site could be used to readily obtain surrogates for currently observed values of *L*_*g*_(*t*, ∆*t*) Several studies show that within our geographical region the parameters *α* and *β* can be considered as time invariant ([1, 11–14]). But even though *α* and *β* are statistically invariant within a given region, environmental influences are expected to induce a relative extent of variability on local estimates of *α* and *β* [11] and as it was stated by Echavarria-Heras et al. [2] this could propagate significant uncertainties on *L*_*ga*_(*α*, *β*, *t*, ∆*t*) values. Indeed the results of Appendix B corroborate that these proxies could produce significantly accurate assessments for *L*_*g*_(*t*, ∆*t*) only in case the fitting of equation (1) yields highly precise estimates of the parameters *α* and *β*.

One important factor contributing to the uncertainty of the estimates of the parameters *α* and *β*, in equation (1) associates to the set of biological influences that could have been disregarded when assuming that eelgrass leaf biomass depends solely on linked length. Another important source of uncertainty relates to the failure of the model of equation (1) to handle environmental effects on a proper way. But the high values of determination coefficients as well as results of residual analysis corresponding to fittings of the model of equation (1) to independent data sets collected in our geographical region ([1, 2, 11, 13, 15, 16]) reveal that on spite of its simplicity, equation (1) is a paradigm that provides highly consistent estimates of the involved parameters.

But beyond uncertainties that biological or environmental influences could convey on estimates of the parameters *α* and *β* in equation (1), there could be other factors affecting their precision. Moreover, the results of Savage et al. [17], Hui and Jackson [18], Packard and Birchard [19], illustrate the relevance of factors like measurement error, data quality, sample size, and regression method on the precision of estimates of the parameters in a bivariate allometric scaling relationship. Moreover, Hui and Jackson [18], Packard and Birchard [19] and Packard and Boardman [20] concluded that in a bivariate allometric scaling nonlinear regression provides better estimates of the allometric exponent than these commonly estimated by linear regressions. Moreover, nonlinear regression was the analysis method used to obtain the significant estimates of the parameters *α* and *β* reported in all the eelgrass studies performed at our geographical region. Therefore, in the present settings we also considered nonlinear regression as an analysis method. However, in comparison with previously reported results ([2, 11, 15]) applying this analysis method to the present data set, resulted in an smaller value for the determination coefficient. Besides, in the current crude data, we detected a proliferation of inconsistent replicates, in the whole showing a noticeable discrepancy to the dispersion pattern that is normally expected for a scaling relationship of the form given by equation (1). Then, we assumed that on the light of the proven consistency of this model, the unusual spreading observed in the present data could on its own; provide an explanation for the reduction in determination coefficient obtained here. This view was strengthened by the fact that the discussed anomalous spreading was not observed for the data sets used in previous analysis of the fitting of a scaling relationship like equation (1) at our study site [11–16]. And, it was perhaps a lack of standardization in processing tasks that led to a relatively larger incidence of measurement error in the present data set. Therefore, we considered that the exploration of data quality effects on the precision of estimates of the parameters *α* and *β* in equation (1) is a fundamental step in the overall procedure that leads to reliable allometric surrogates for the assessment of eelgrass leaf growth rates. Since, the aforesaid study had not been yet done; we made here an attempt to fill this gap.

Eelgrass leaf biomass is lognormally distributed, and the sizes of the different groups of replicates that make up our data are reduced. Therefore, we removed inconsistent replicates from the set of crude data by using a robust median absolute deviations cleaning procedure. We also relied on a sensitivity analysis study in order to assess the improvement in the accuracy of the *L*_*ga*_(*α*, *β*, *t*, ∆*t*) proxy that may possibly be associated to whatever reduction on the uncertainties of *α* and *β* could be derived from an improvement in data quality. Besides, we explored the corresponding effects of sample size, by randomly drawing differently sized samples out of both the crude and the processed data sets and then comparing the values of the determination coefficients for the fittings of equation (1) to the selected samples. In the Methods section we further substantiate the data cleaning approach, the procedure for estimating the effects of sample size as well as the steps of the performed sensitivity analysis. In the Results and Discussion section we elaborate on the relevance and limitations of this study. In the Conclusions section we presented the summary and potential implications of our findings.

## Methods

### Raw data

For the aims of the present research we assembled an extensive data set containing measurements taken on a total of 10412 individual eelgrass leaves collected in San Quintin Bay Baja California as previously reported in ([1, 2, 11–14]). Crude data includes measurements of length (mm), width (mm) and dry weight (gr).

### Data quality control procedures

**Fig. 1:**
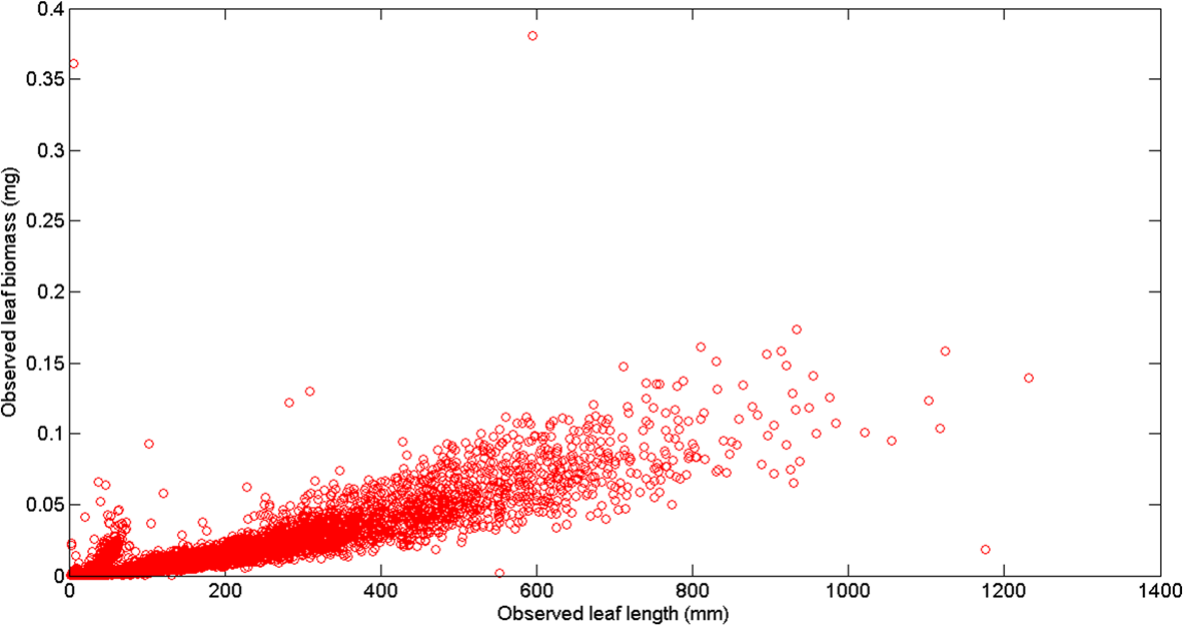
Observed leaf length and biomass values. A plot of leaf length versus leaf biomass data reveals a power function-like trend masked by a great variability in observed leaf weight replicates for a given leaf size. Departure from an expected power function-like trend is more pronounced for smaller and larger leaves. The dispersion pattern shown suggests that data processing errors might have induced unduly biased leaf biomass values

Short [21] and Gaeckle and Short [22] estimated leaf biomass in eelgrass by using an isometric weight to length ratio, and the appropriateness of allometric methods in eelgrass research has been validated for a number of independent data sets (e.g. [1, 2, 11–16] and references therein). Particularly, Echavarria-Heras et al. [2] formally demonstrated that the conspicuous leaf architecture and growth form of eelgrass makes leaf length a reliable allometric descriptor of the associated biomass. Indeed, results show that for independent data sets collected in our geographical region the model of equation (1) always produced reliable fits ([1, 2, 11–13, 15, 16]). Moreover, the results of Solana-Arellano et al. [11] stating that the parameters associated to the scaling relationship of equation (1) can be considered invariant within a given geographical region, endorse that this model is highly consistent. This makes it reasonable to assume that the true relationship linking *w*(*t*) and *l*(*t*) should conform to a power function like dependency and consequently we could expect a typical dispersion for the linked data. In other words, the inherent spreading pattern should fit in a dominant power function trend masked by expected stochastic variability induced by intrinsic biological factors and environmental forcing. Particularly, at our study site the fitting of equation (1) produced a determination coefficient of *R*^2^ = 0.90 for the data set addressed in [2]. Meanwhile, for data set in [11] as well as for that reported in [15] the corresponding value was *R*^2^ = 0.92, with consistency of residuals verified for all these fits. Since in comparison with these results, for the present set of crude data the fitting of the model of equation (1) produced a reduced determination coefficient (*R*^2^ = 0.81) we concluded that differences in determination coefficients could be explained by inconsistencies in the present data that could be linked to factors beyond biological influences or local environmental forcing. Moreover, by using a direct survey of the dispersion pattern shown by the present crude data (Fig. 1) we observed that departure of a given replicate from an expected power function-like trend was more pronounced for leaves with smaller sizes. Since, this effect was not observed in related data sets collected in our region, we judged that these inconsistencies could be in a fundamental way tied to the considerably larger extension of the present data set, which required processing performed by several technicians and it was perhaps a lack of standardization in these tasks that explains the present proliferation of inconsistencies that should be removed in order to prevent their permanence from exerting a reduction in the precision of estimates for the parameters in the scaling relationship of equation (1). Notwithstanding, the judgment to remove an outlier or inconsistent measurement in a data set, it is necessary to be able to identify its occurrence. Data cleaning procedures tasks are commonly performed by using a mean plus or minus three standard deviations method. This technique is based on the property of a normal distribution for which 99.87 % of the data appear within this range [23], but its application in the present settings presents difficulties. It firstly assumes that the distribution of data is normal while eelgrass leaf biomass is lognormally distributed [2]. Secondly, both the mean and standard deviation are strongly impacted by outliers [24]. Thirdly, our data is composed by groups of measurements that include a reduced number of leaf biomass replicates, and the mean plus or minus three standard deviations method is very unlikely to detect outliers in small samples [25]. This fact made it also inconvenient to use alternative data cleaning techniques like studentized residuals, the hat elements, Cook’s distance, or the Mahalanobis distance procedure [26]. Alternatively, the median of a group of data is totally immune to the sample size and a robust estimator of scale, reason why we adapted a Median Absolute Deviation (MAD) method [27] which provided the present criteria for the removal of inconsistencies in groups of more than 10 replicates of biomass values associated to a given leaf length. By eliciting the consistency of the allometric scaling of equation (1), we assumed that the addressed length-to-weight variation pattern should conform to a power function-like trend Therefore, individual leaf biomass measurements that in an initial data analysis were found to severely deviate from this pattern in sets under 10 replicates were removed from the set of unprocessed data. In summary, by analyzing the spreading of leaf biomass values in a leaf length-to-weight plot, we detected replicates that we supposed represented unduly deviations from an expected power function-like variation pattern. By applying the explained data cleaning procedures we removed these from the set of crude data, because in light of the proven consistency of the scaling relationship of equation (1) these inconsistent replicates could have been caused by errors due to a lack of standardization in leaf length or biomass measurements, or perhaps, explained by errors owed to faulty equipment for dry weight assessment or even explained by incorrect recordings.

In order to formalize the present MAD data cleaning procedure, we arranged the crude data in different groups *G*(*l*) = {*w*_*G*1_(*l*), …, *w*_*Gn*_(*l*)} formed by an observed leaf length *l* and associated leaf biomass replicates *w*_*G*1_(*l*), …, *w*_*Gn*_(*l*). For almost all groups of replicates *G*(*l*) we observed several leaf biomass values that parted from the expected variability pattern and were considered as inconsistencies. For each group *G*(*l*) we firstly obtained its median denoted by means of the symbol *MED*{*w*_*G*1_(*l*), …., *w*_*Gn*_(*l*)} or simply by means of *M*(*G*(*l*)) for short. Then, for each replicate *w*_*Gj*_(*l*) in (*G*(*l*) we calculated its absolute deviation from the group median δ_*Gj*_(*l*), that is, *δ*_*Gj*_(*l*) = |*w*_*Gj*_(*l*) − *M*(*G*(*l*))|. Similarly, we obtained the median of the set of absolute deviations denoted by the symbol *MED*{ *δ*_*G*1_(*l*), …, *δ*_*Gn*_(*l*)}. Following, Huber [28] and also recalling that eelgrass leaf biomass values are log-normally distributed [2], we obtained the Median Absolute Deviation of a group *G*(*l*) denoted here through *MAD*(*G*(*l*)) and given by

2 $$ MAD\left(G(l)\right)= bMED\left\{\ {\delta}_{G1}(l),\dots, {\delta}_{Gn}(l)\right\}, $$

where *b* = 1/*Q* (0.75), being *Q* (0.75) the 0.75 quantile of the lognormal distribution.

For the removal of inconsistent replicates in a group *G*(*l*) we used the decision criterion

3 $$ M\left(G(l)\right)-T\cdot MAD\left(G(l)\right)<{w}_j(l)<M\left(G(l)\right)\kern0.5em +T\cdot MAD\left(G(l)\right), $$

where *T* is the rejection threshold that following Miller [24], we set at a value of *T* = 3. For groups *G*(*l*) under ten replicates we applied a direct data cleaning procedure by removing replicates that we considered were severely deviated from the central power function-like trend.

### Sensitivity analysis

In order to evaluate the influences that the uncertainties of the parameters *α* and *β* convey in the performance of *L*_*ga*_(*α*, *β*, *t*, ∆*t*), we used both a formal study and also simulation runs. The analytical exploration is presented in Appendix B. For the simulation study, initially, at equally spaced iteration (i.e., sampling) times *t*, we drawn uniformly distributed random numbers representing a number of shoots retrieved *NS* (*t*, ∆*t*) and the number *nl*(*s*) of leaves that each one of these shoots holds. Afterwards, the lengths *l*(*t* + ∆*t*) of each one the leaves in a shoot were simulated according to a fitted lognormal distribution, (*p* > 0.05), with a mean of 207.24 mm and a standard error of 3.8 [2]. Similarly, the corresponding leaf length increments, ∆*l*, were also extracted from a fitted lognormal distribution (*p* > 0.05) with a mean of 140.04 mm and a standard error of 7.3 [2]. The values for these increments coincided with the length of the whole leaf, as it occurs in newly produced leaves. Subsequently, we utilized the simulated leaf lengths and estimates $\widehat{\alpha}$ and $\widehat{\beta}$ of the parameters *α* and *β* in equation (1), in order to produce the proxy growth rates *L*_*ga*_(*α*, *β*, *t*, ∆*t*). In order to produce estimates $\widehat{\alpha}$ and $\widehat{\beta}$ for the parameters *α* and *β* we followed Hui and Jackson [18], Packard and Birchard [19] and Packard and Boardman [20] and fitted equation (1) to the present data sets using an iterative nonlinear least-squares method rather than the traditional approach of linearizing the equation through a logarithmic transformation of data. All fittings were performed using the Matlab Statistics Toolbox.

To study the sensitivity of *L*_*ga*_(*α*, *β*, *t*, ∆*t*) to changes in the parameters *α* and *β* we selected as reference values the estimates $\widehat{\alpha}$ and $\widehat{\beta}$ and produced changing values α_*ep*_ for $\widehat{\alpha}$ and *β*_*eq*_ for $\widehat{\beta}$ namely,

4 $$ {\alpha}_{ep} = \widehat{\alpha}+\varDelta {\alpha}_p, $$

5 $$ {\beta}_{eq}=\widehat{\beta}+\varDelta {\beta}_q, $$

with the values ∆α_*p*_ and ∆α_*q*_ satisfying

6 $$ \left|\varDelta {\alpha}_p\right| = p\cdot stde\left(\widehat{\alpha}\right) $$

and

7 $$ \left|\varDelta {\beta}_q\right| = q\cdot stde\left(\widehat{\beta}\right), $$

where $stde\left(\widehat{\alpha}\right)$ and $stde\left(\widehat{\beta}\right)$ stand for the standard errors of $\widehat{\alpha}$ and $\widehat{\beta}$ respectively and *p* and *q* are numbers satisfying, 0 < *p* ≤ 1 and 0 < *q* ≤ 1.

Therefore, the fluctuating values *α*_*ep*_ and *β*_*eq*_ for $\widehat{\alpha}$ and $\widehat{\beta}$ are scaled through proportions *p* and *q* of their standard errors. In our analysis we also relied on a parameter change index denoted by means of *ρ*(*p, q*), and defined through

8 $$ \rho \left(p,\ q\right) = \sqrt{{\left(\widehat{\beta}-{\beta}_{eq}\right)}^2+{\left(\widehat{\alpha}-{\alpha}_{ep}\right)}^2}. $$

Particularly, the maximum value that *ρ*(*p, q*) can attain as *p* and *q* vary is denoted through the symbol *ρ*_*max*_(*p*, *q*) and is given by,

9 $$ {\rho}_{max}\left(p,q\right) = \sqrt{ste{\left(\widehat{\alpha}\right)}^2+ste{\left(\widehat{\beta}\right)}^2\kern0.75em } $$

In what follows we will also use the mean value of *ρ*(*p, q*) denoted here by *ρ*_*av*_(*p, q*) and given by

10 $$ {\rho}_{av}\left(p,q\right)=\sqrt{stde{\left(\alpha \right)}^2+ stde{\left(\beta \right)}^2/2}. $$

The simulated leave lengths *l*(*t + ∆t*), corresponding increments ∆*l* the *NS*(*t*, ∆*t*) and *nl*(*s*) values, and the parameter estimates $\widehat{\alpha}$ and $\widehat{\beta}$ produced by means of equation (17) a reference trajectory ${L}_{ga}\left(\widehat{\alpha}, \widehat{\beta},t,\varDelta, t\right)$. It turns out that every picked value of *ρ*(*p, q*) yields different pairs (*α*_*ep*_, *β*_*eq*_), each one associated to a couple (∆*α*_*p*_, ∆*β*_*q*_) that comply with the condition of equation (8). For each pair(*α*_*ep*_, *β*_*eq*_), the simulated leave and shoot data and equation (17) shape a trajectory *L*_*ga*_(*α*_*ep*_, *β*_*eq*_, *t*, ∆*t*). The average of the values that these trajectories produce at each sampling time *t* is then calculated and its value denoted through 〈*L*_*ga*_(*α*_*ep*_, *β*_*eq*_, *t*, *Δt*)〉_*ρ*_. Following the procedure, requires to calculate the deviations between 〈*L*_*ga*_(*α*_*ep*_, *β*_*eq*_, *t*, *Δt*)〉_*ρ*_ and ${L}_{ga}\left(\widehat{\alpha}, \widehat{\beta},t,\varDelta t\right)$, that are denoted by means of the symbol *δL*_*gaρ*_(*α*_*ep*_, *β*_*eq*_, *t*, *Δt*) and obtained through,

11 $$ \delta {L}_{ga\rho}\left({\alpha}_{ep},{\beta}_{eq},,,t,,\varDelta t\right)={\left\langle {L}_{ga}\left({\alpha}_{ep},{\beta}_{eq},,t,\varDelta t\right)\right\rangle}_{\rho }-{L}_{ga}\left(\widehat{\alpha},\ \widehat{\beta},t,\varDelta t\right). $$

We also need to compute the average through time of the *δL*_*gaρ*_(*α*_*ep*_, *β*_*eq*_, *t*, *Δt*) deviations whose output is represented here through the symbol 〈*δL*_*gaρ*_(*α*_*ep*_, *β*_*eq*_, *t*, *Δt*)〉_*t*_. Finally, it is necessary to obtain the time average of the values of the reference trajectory ${L}_{ga}\left(\widehat{\alpha}, \widehat{\beta},t,\varDelta t\right)$. The resulting record is denoted using the symbol ${\left\langle {L}_{ga}\left(\widehat{\alpha}, \widehat{\beta},t,\varDelta t\right)\right\rangle}_t$. These statistics are then used to produce the relative deviation index *ϑ*(*Δα*_*p*_, *Δβ*_*q*_)_*ρ*_ given by

12 $$ \vartheta {\left(\varDelta {\alpha}_p,\ \varDelta {\beta}_q\ \right)}_{\rho }=\frac{\left|{\left\langle \delta {L}_{ga\rho}\left({\alpha}_{ep},\ {\beta}_{eq},t,\varDelta t\right)\right\rangle}_t\right|\kern0.5em }{{\left\langle {L}_{ga}\left(\widehat{\alpha},\ \widehat{\beta},t,\varDelta t\right)\right\rangle}_t}. $$

The value of *ϑ*(*Δα*_*p*_, *Δβ*_*q*_)_*ρ*_ provides a measure of the sensitivity of the reference trajectory ${L}_{ga}\left(\widehat{\alpha}, \widehat{\beta},t,\varDelta t\right)$ to a change of tolerance *ρ*(*p, q*) on the pair ($\widehat{\alpha}$, $\widehat{\beta}$). Moreover, by calculating *ϑ*(*Δα*_*p*_, *Δβ*_*q*_)_*max*_ defined through,

13 $$ \vartheta {\left(\varDelta {\alpha}_p,\ \varDelta {\beta}_q\right)}_{max} = {max}_{\rho}\left\{\vartheta {\left(\varDelta {\alpha}_p,\ \varDelta {\beta}_q\right)}_{\rho}\right\}, $$

we can get in what percentage of ${\left\langle {L}_{ga}\left(\widehat{\alpha}, \widehat{\beta},t,\varDelta t\right)\right\rangle}_t$ the maximum absolute deviation between *L*_*ga*_(*α*_*ep*_, *β*_*eq*_, *t*, *Δt*) and ${L}_{ga}\left(\widehat{\alpha}, \widehat{\beta},t,\varDelta t\right)$ amounts.

### Sample size effects

In order to explore the extent of sample size influences on ${L}_{ga}\left(\widehat{\alpha}, \widehat{\beta},t,\varDelta t\right)$ we performed the fitting of equation (1), using differently sized samples of leaf length and biomass (*l*(*t*),*w*(*t*)) data which were randomly drawn from the available datasets. For each sample of size *n*, we obtained the determination coefficient (*r*_*n*_^2^) as well as the values of the estimators of the parameters ${\widehat{\alpha}}_n$ and ${\widehat{\beta}}_n$, their respective standard errors $std{e}_n\left(\widehat{\alpha}\right)$ and $std{e}_n\left(\widehat{\beta}\right)$ and the values of the upper bound *ρ*_*max*_(*p*, *q*)_*n*_ and *ϑ*(*Δα*_*p*_, *Δβ*_*q*_ )_*ρmax*_.

## Results and discussion

In seagrass research, allometric equations have provided useful empirical models aimed to the representation of biologically relevant traits *Y* in terms of an easily measured variable *X* (e.g. [29–33]). This is readily exemplified by equation (1) that provides a convenient representation of eelgrass leaf biomass *w*(*t*) in terms of associated length *l*(*t*). But the realm of allometric methods in eelgrass research is not only limited to empirical formulations that provide convenient proxies for leaf biomass estimations. Indeed from a theoretical standpoint, allometric approaches have provided for instance, a framework for the evaluation of the suitability of the leaf-biomass-to length ratio as a tool for nondestructive leaf biomass evaluations [15] and have also endorsed the substantiation of the plastochrone method of leaf growth assessments [16]. Moreover, equation (1) is the foundation of the derivation of the *L*_*ga*_(*α*, *β*, *t*, Δ*t*) proxy of equation (17) a paradigm aimed to the indirect assessment of eelgrass leaf growth rates ([1, 2, 12]).

The effectiveness of the *L*_*ga*_(*α*, *β*, *t*, ∆*t*) construct for providing truly accurate and non-destructive assessments for the observed average rate of leaf growth per shoot-day *L*_*g*_(*t*, ∆*t*) depends on both, the time invariability of the parameters *α* and *β*, and on the accuracy of their estimates. From a general standpoint, the variability of the allometric exponent $\alpha$ has been the focus of theoretical and empirical studies because it often seems to have a constant value specific to a particular biological relationship (e.g. [34–39]). On the other hand, several studies have provided evidence that support certain variability in the exponent of allometric scaling laws (e.g. [40–45]). Accordingly, the value of the normalization constant $\beta$ is thought to be characteristic of species or populations [46]. And the variability observed in the normalization constant, explained as a differential response to environmental conditions [34, 35, 47–49]. Particularly, for eelgrass, Solana-Arellano et al. [11] analyzed independent data set collected in different geographical regions to conclude that no universal values can be found for the allometric parameter α in equation (1), suggesting as well that this scaling relationship might be considered static, thus implying that local factors determine the extent of the variability of the actual values of the parameters *α* and *β*. On the presence of this variability associated uncertainties are expected to spread inaccuracies in eelgrass leaf growth rates produced by means of equation (17).

**Fig. 2:**
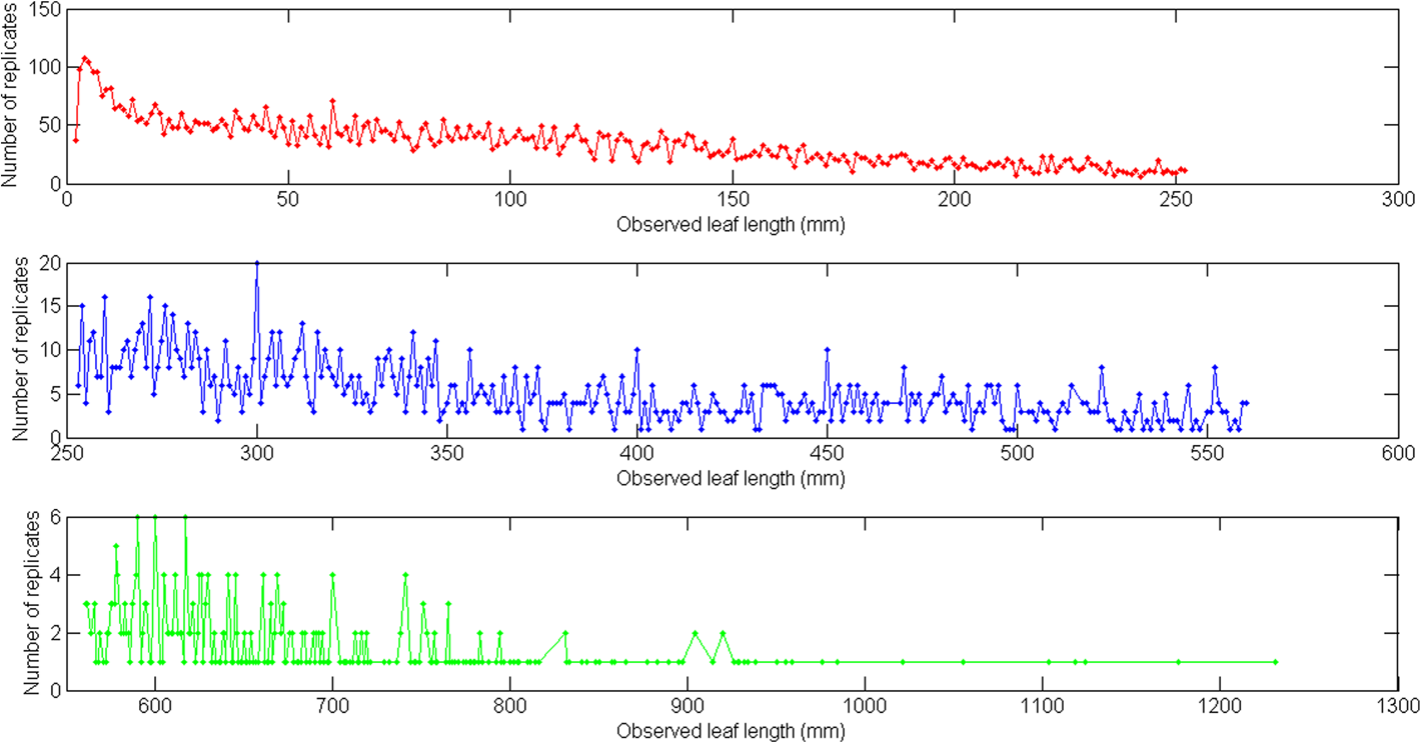
Leaf lengths and numbers of associated leaf biomass replicates. The distribution of the number of replicates of leaf biomasses for a given length decreases with leaf length. Maximum number of replicates was 107 and corresponded to a leaf length of 3 mm

**Fig. 3:**
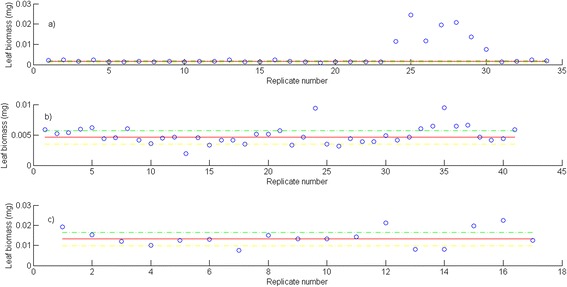
Examples of the distribution of leaf biomass replicates for a given leaf length. **a** Leaf biomass replicates linked to *l* = 50 mm, (**b**) Leaf biomass replicates corresponding to *l* = 101 mm, and (**c**) Leaf biomass replicates associated to *l* = 200 mm. In these plots red lines stand for the group median: *M*(*G*(*l*)), yellow lines represent the *M*(*G*(*l*)) - 3∙*MAD*(*G*(*l*)) threshold and green lines show the *M*(*G*(*l*)) + 3∙*MAD*(*G*(*l*) threshold

**Fig. 4:**
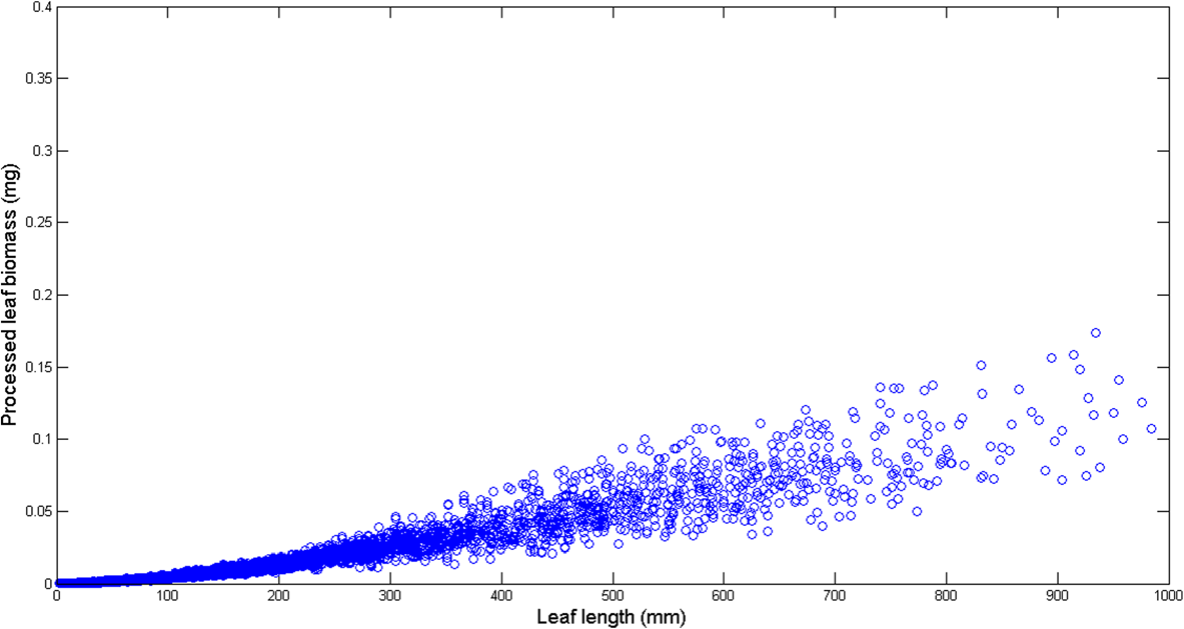
Plot of quality controlled data. This plot shows the distribution of replicates of eelgrass leaf biomass that remained after applying the addressed quality control procedures. About 30 % of replicates in the raw data set were found inconsistent

**Fig. 5:**
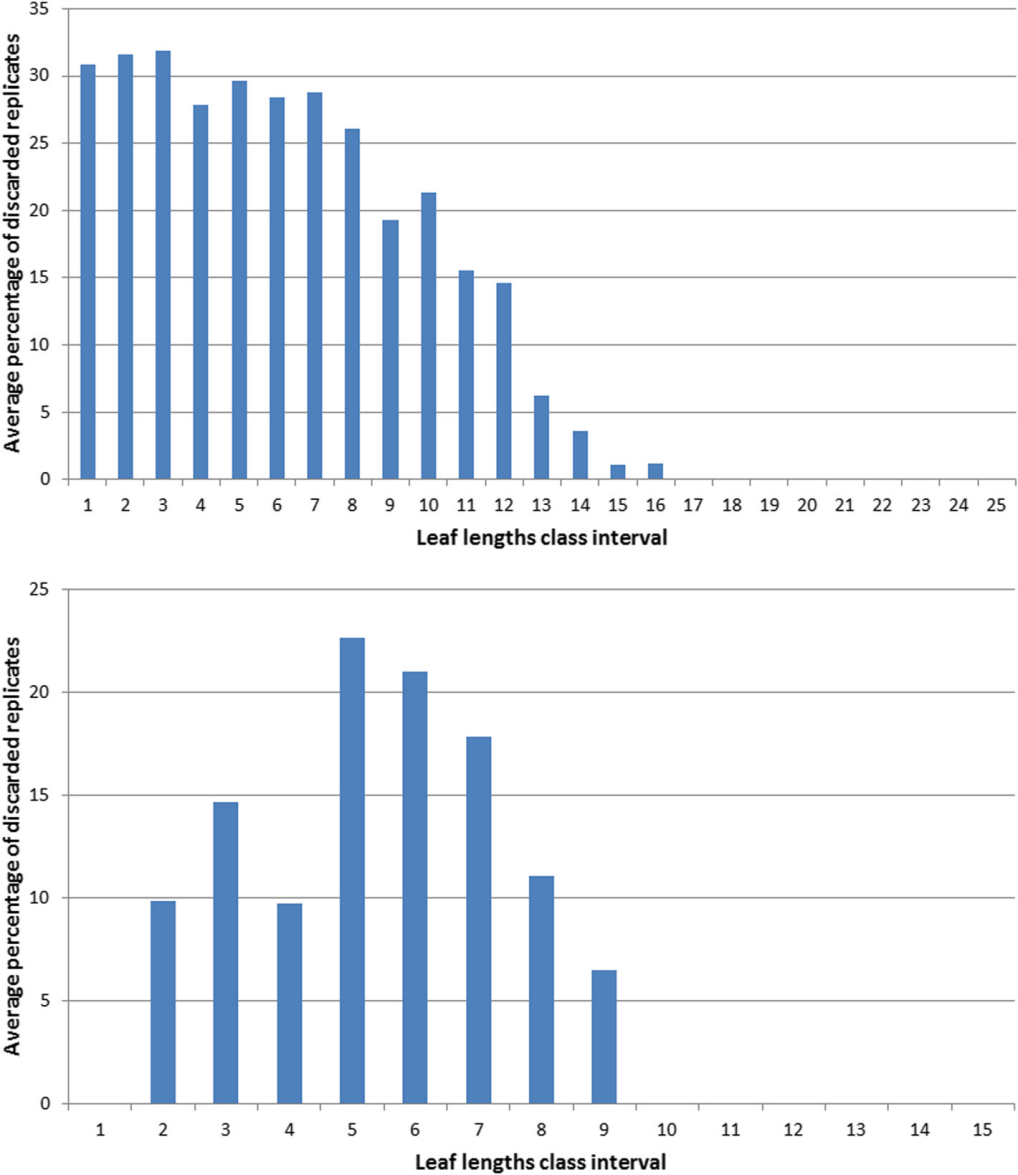
Comparison of results of present data cleaning procedures applied to the current and an independent data set. This figure exhibits the results of applying the present data cleaning procedures applied to the current data and another equivalent data set reported in [12]. For both data sets we formed leaf length class intervals covering the extent of observed values, with the extension of each class interval set at 50 mm. The first length class interval is labeled by 1 and represents the set of leaf length lying within interval 0 < *l*(*t*) ≤ 50. Class 2 associates to the interval 50 < *l*(*t*) ≤ 100 and so one (horizontal axis). For each set of replicates associated to a given length in a class interval we obtained the percentage of removed inconsistencies, then for each class interval we obtained the average of these percentages (vertical axis). For all of the considered length class intervals we observed relatively greater average percentages of data inconsistencies in the present crude data

Figure 1 displays the variability of observed leaf lengths and linked biomasses. The survey of the crude data reveals a notorious proliferation of replicates associated to an observed leaf length. Data was arranged into 755 different groups *G*(*l*) = {*w*_*G*1_(*l*),…,*w*_*Gn*_(*l*)}. Figure 2 displays the variation of the number of replicates for a given leaf size. We can observe that the number of replicates in groups *G*(*l*) decreases with leaf size. This is probably due to the fact that separation from shoots by drag forces is more pronounced for longer and older leaves than it is for small and younger leaves. Figure 3 shows examples of the dispersion within *G*(*l*) groups. Moderated differences in replicated leaf biomass values associating to a given length can be explained by the inherent stochastic variability along with normal systematic errors introduced by data processing. Nevertheless, an exploration of the dispersion pattern of leaf biomass values in the present crude data reveals replicates representing unduly deviations from the expected power function-like trend. These inconsistent measurements are more visible for leaves under 100 mm long and also for those longer than 800 mm. For groups with 10 or more replicates and according to the present MAD data quality control criterion, whenever an individual leaf biomass replicate *w*_*Gj*_(*l*) differed in magnitude from the group median by more than 3 times the MAD it was considered as an inconsistency and it was rejected [50]. Similarly, the power function-like trend data cleaning criteria described in the methods section permitted the removal of inconsistent leaf biomass values in groups with less than 10 replicates. Figure 4 presents a plot of the leaf biomass values that remained after applying data quality control. This procedure retained a representative number of replicates showing the normally expected variability of eelgrass leaf biomass values ([1, 2, 11–16]). Moreover, in order to exemplify differences in quality among equivalent data sets we applied the present data cleaning procedure to equivalent data reported in Echavarria-Heras et al. [12]. For easy of presentation we chose leaf length class intervals covering the extent of observed leaf lengths in both data sets. For each one of the lengths of in these class intervals we applied the present MAD procedure and calculated the percentage of discarded replicates. Then we obtained the average percentage of discarded leaves that associate to each length class interval. Figure 5 compares the average percentages of discarded replicates per leaf length class for the present data and for the Echavarria-Heras et al. [12] data set. For leaf lengths lying in the first class interval (0 ≤ *l*(*t*) < 50) the average percentage of rejected replicates in the present data set was 30 % while the Echavarria-Heras et al. [12] data reported no inconsistent replicates for the same interval. And generally, the percentages of discarded replicates in the present data set were markedly bigger than those associated to the Echavarria-Heras et al. [12] data. This comparison can explain why the fitting the model of equation (1) to the present crude data set produced an smaller determination coefficient than that reported in Echavarria-Heras et al. [12].

Observations show that the order relationship 0 < *β* < *α* holds [1, 2, 11, 12] and that it is also reasonable to consider the variation ranges of ∆*α* and ∆*β* satisfying: |∆*α*| < *α* and |∆*β*| < *β*. By embracing these assumptions in Appendix B we derived explicit formulae for the deviations *δL*_*ga*_(*Δα*, *Δβ*, *t*, *Δt*) that changed values of the parameters *α* and *β* having the form *α*_*e*_ = *α* + ∆*α* and *β*_*e*_ = *β* + ∆*β* induce in the reference values *L*_*ga*_(*α*, *β*, *t*, ∆*t*). These are given by equations (21). By assuming that the observed order relationship for the parameters *α* and *β* holds and also by considering the aforesaid expected variation ranges for ∆*α* and ∆*β*, we found that the proxies *L*_*ga*_(*α*, *β*, *t*, ∆*t*) for the average rate of leaf growth per shoot-day, will be overestimated by *L*_*ga*_(*α*_*e*_, *β*_*e*_, *t*, ∆*t*) values, primarily when the ordered pair (∆*α*, ∆*β*) lies on the domain ∆*α* > 0 and ∆*β* > 0. Nevertheless, as it is explained in Appendix B, each positive value of ∆*β*, can be associated to a set of negative values of ∆*α* for which *L*_*ga*_(*α*, *β*, *t*, ∆*t*) will be also overestimated by *L*_*ga*_(*α*_*e*_, *β*_*e*_, *t*, ∆*t*). Correspondingly, *L*_*ga*_(*α*_*e*_, *β*_*e*_, *t*, ∆*t*) underestimates *L*_*ga*_(*α*, *β*, *t*, ∆*t*) whenever the pair (∆*α*, ∆*β*) is placed inside the region -*α* < ∆*α* < 0 and -*β* < ∆*β* < 0, but this time, each value of ∆*β* satisfying -*β* < ∆*β* < 0 can be associated to a set of positive values of ∆*β* for which *L*_*ga*_(*α*, *β*, *t*, ∆*t*) will be also underestimated by *L*_*ga*_(*α*_e_, *β*_e_, *t*, ∆*t*).

**Fig. 6:**
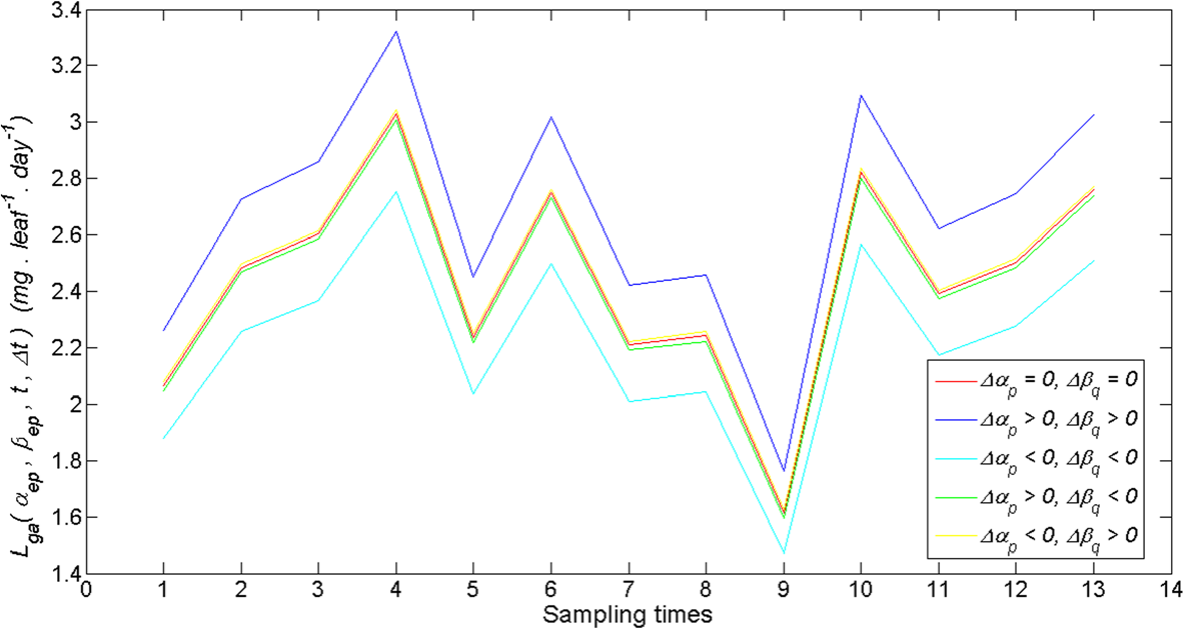
Examples of deviations between allometrically projected leaf growth rates. We portrait several trajectories of allometrically projected leaf growth rates *L*_*ga*_(*α*_*ep*_, *β*_*eq*_,*t*, ∆*t*) in units of mg. leaf ^−1^.day^−1^. These trajectories were produced by variations *α*_*ep*_ and *β*_*ep*_ in fitted parameters $\widehat{\alpha}$ and $\widehat{\beta}$, complying with the condition *ρ*(*p*, *q*)^2^ = *stde*($\widehat{\alpha}$)^2^ + *stde*($\widehat{\beta}$)^2^/4. We show the reference trajectory ${L}_{ga}\left(\widehat{\alpha},\ \widehat{\beta},t,\varDelta t\right)$ in red. The number of days elapsed between sampling times is 15

**Fig. 7:**
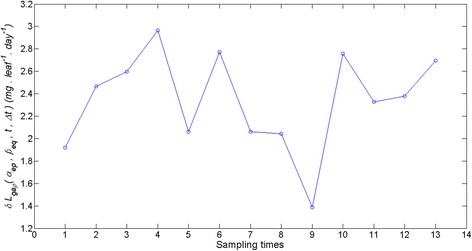
Example of the trajectory of deviations *δL*_*gaρ*_(*α*_*ep*_, *β*_*eq*_, *t*, *Δt*). We show an example of the behavior of the trajectory of deviations *δL*_*gaρ*_(*α*_*ep*_, *β*_*eq*_, *t*, *Δt*). This is obtained by subtracting at each time *t*, the value of the reference trajectory ${L}_{ga}\left(\widehat{\alpha},\ \widehat{\beta},t,\varDelta t\right)$ from the average of the values of the different trajectories *L*_*ga*_(*α*_*ep*_, *β*_*eq*_, *t*, *Δt*) produced by a fixed value of the parameter change index *ρ*(*p*, *q*). This average is denoted by means of the symbol 〈*L*_*ga*_(*α*_*ep*_, *β*_*eq*_, *t*, *Δt*)〉_*ρ*_ (cf. Equation (11)). The number of days between sampling times is 15

**Fig. 8:**
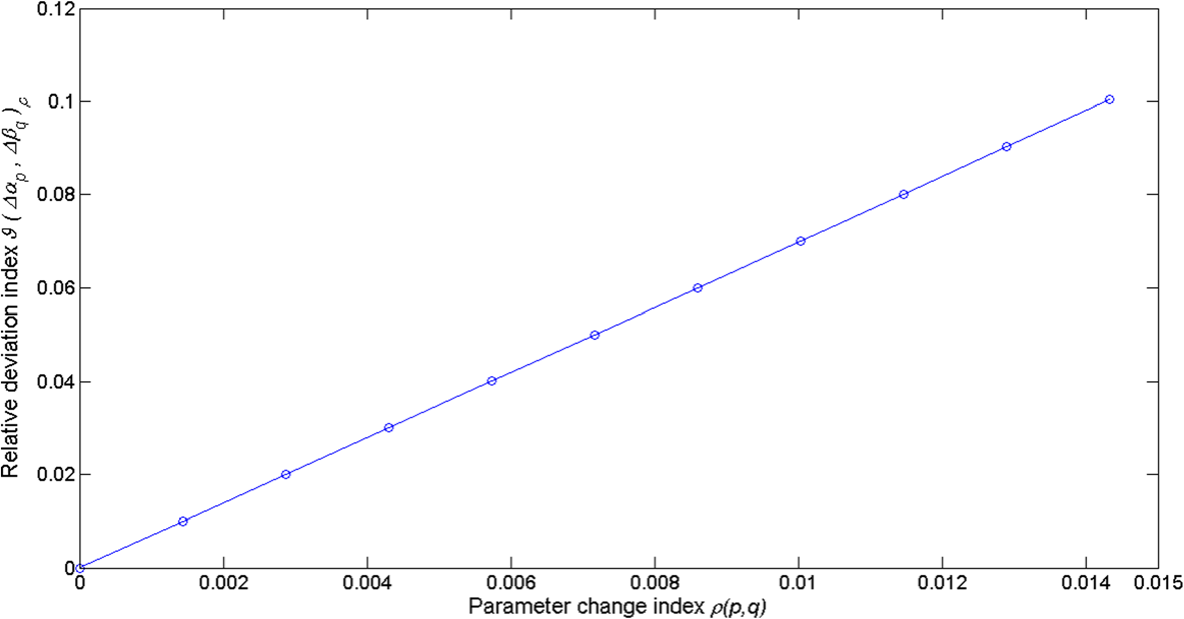
The behavior of the relative deviation index *ϑ*(*Δα*_*p*_, *Δβ*_*q*_ )_*ρ*_. This plot shows the behavior of the relative deviation index for different values of *ρ*(*p*, *q*). This index *ϑ*(*Δα*_*p*_, *Δβ*_*q*_ )_*ρ*_ is obtained by dividing the absolute value of the average through time of the deviations between the 〈*L*_*ga*_(*α*_*ep*_, *β*_*eq*_, *t*, *Δt*)〉_*ρ*_ and ${L}_{ga}\left(\widehat{\alpha},\ \widehat{\beta},t,\varDelta t\right)$ rates, by the time average of the ${L}_{ga}\left(\widehat{\alpha},\ \widehat{\beta},t,\varDelta t\right)$ values, (cf. Equation (12)). The relative deviation index *ϑ*(*Δα*_*p*_, *Δβ*_*q*_ )_*ρ*_ provides a measure of the sensitivity of the allometric method *L*_*ga*_(*α*, *β*, *t*, *Δt*) to changes in the parameters *α* and *β*. The values of the relative deviation index *ϑ*(*Δα*_*p*_, *Δβ*_*q*_ )_*ρ*_ are bounded above by a value *ϑ*(*Δα*_*p*_, *Δβ*_*q*_ ) _*max*_ = 0.01. (cf. Equation (13))

**Fig. 9:**
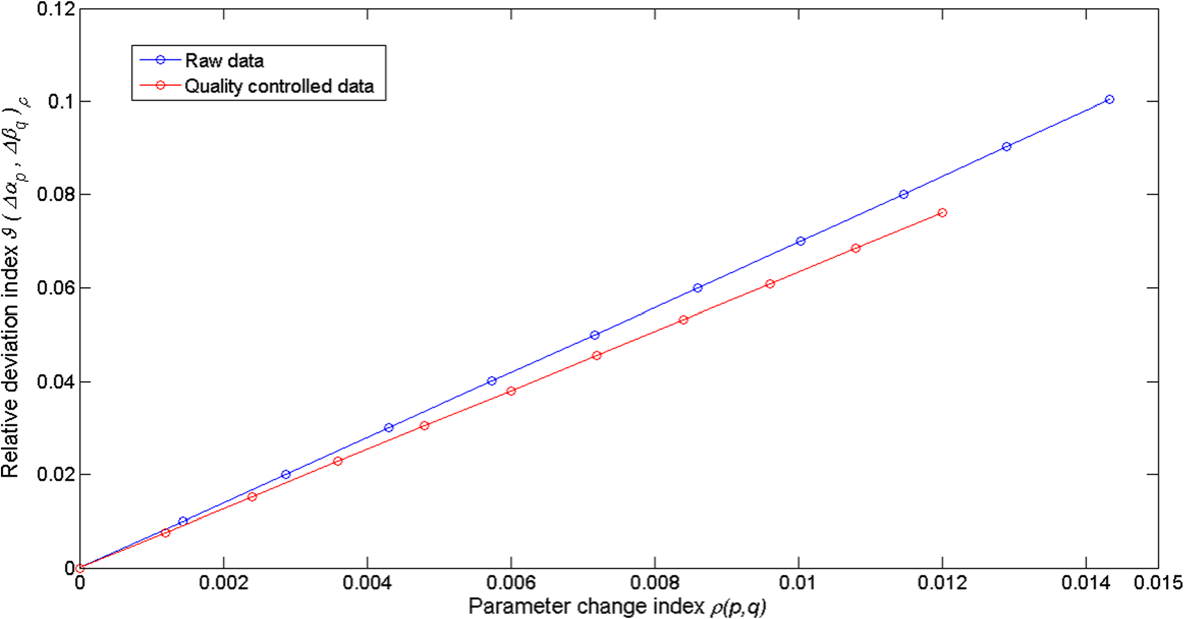
Comparison of relative deviation index for crude and quality controlled data. This plot compares the values for the relative deviation index *ϑ*(*Δα*_*p*_, *Δβ*_*q*_ )_*ρ*_ calculated as the parameter change index *ρ*(*p*, *q*) varies. Values of *ϑ*(*Δα*_*p*_, *Δβ*_*q*_ )_*ρ*_ produced by crude data are shown using blue lines and those corresponding to quality controlled data in red lines

By fitting of equation (1) to the crude leaf length and weight data we obtained *r*^2^ = 0.81 and estimates, $\widehat{\alpha}=1.32367$ with $stde\left(\widehat{\alpha}\right) = 0.0143$ for *α* and of $\widehat{\beta}=0.000014$ with $stde\left(\widehat{\beta}\right)=1.4e-6$ for *β*. Therefore, according to equation (8) we have 0 ≤ *ρ*(*p*, *q*) ≤ 0.0143. Using the $\widehat{\alpha}$ and $\widehat{\beta}$ estimates we obtained *ρ*_*max*_(*p*, *q*) = 0.0143 (cf. Eq. (9)) and *ρ*_*av*_(*p*, *q*) = 0.0072 (cf. Eq. (10)). By means of the simulated leave and shoot data and equation (17) we estimated the ${L}_{ga}\left(\widehat{\alpha},\ \widehat{\beta},t,\varDelta t\right)$ reference trajectory. Afterwards for fixed values of the parameter change index *ρ*(*p*, *q*) we used equations (6) and (7) to produce values for the ∆*α*_*p*_ and ∆*β*_*p*_ increments complying with the condition of equation (8). This allowed the characterization of the associated *L*_*ga*_(*α*_*ep*_, *β*_*eq*_, *t*, ∆*t*) trajectories. Figure 6 displays trajectories obtained for the case in which *ρ*(*p*, *q*) takes its average value *ρ*_*av*_(*p*, *q*). These simulation outputs are consistent with the results of the appendix setting domains of variation of the pair (∆*α*, ∆*β*) where *L*_*ga*_(*α*, *β*, *t*, ∆*t*) values are overestimated or underestimated. Similarly, Fig. 7 shows the time variation of the average deviations 〈*δL*_*ga*_(*Δα*_*p*_, *Δβ*_*q*_, *t*, *Δt*)〉_*ρ*_ obtained for *ρ*(*p*, *q*) = *ρ*_*av*_(*p*, *q*). Moreover, Fig. 8 shows that whenever 0 ≤ *ρ*(*p*, *q*) ≤ *ρ*_*max*_(*p*, *q*) the values of the relative deviation index *ϑ*(*Δα*_*p*_, *Δβ*_*q*_)_*ρ*_ are bounded above by a value *ϑ*(*Δα*_*p*_, *Δβ*_*q*_) _*max*_ = 0.01, that is, the maximum absolute deviation between *L*_*ga*_(*α*_*ep*_, *β*_*eq*_, *t*, ∆*t*) and ${L}_{ga}\left(\widehat{\alpha},\ \widehat{\beta},t,\varDelta t\right)$ amounts to 10 % of ${\left\langle {L}_{ga}\left(\widehat{\alpha},\ \widehat{\beta},t,\varDelta t\right)\right\rangle}_t$. In turn, when fitting of equation (1) to the quality controlled data we obtained *r*^2^ = 0.91 and estimates $\widehat{\alpha}=1.403$ with $stde\left(\widehat{\alpha}\right)=0.0120$ for *α* and of $\widehat{\beta}=8.462e-006$ with $stde\left(\widehat{\beta}\right) = 6.4300e-007$, for *β*. Therefore, according to equation (9) this time we have *ρ*_*max*_(*p*,*q*) = 0.01. In turn, performing the sensitivity study of equations (4) through (13) we obtained *ϑ*(*Δα*_*p*_, *Δβ*_*q*_)_*max*_ = 0.07, which amounts to 7 % of ${\left\langle {L}_{ga}\left(\widehat{\alpha},\ \widehat{\beta},t,\varDelta t\right)\right\rangle}_t$ (see Fig. 9).

This sensitivity exploration, sets the accuracy of the allometric method of equation (17) to be mainly dependent on the extent of the upper bound *ρ*_*max*_(*p*, *q*) of the parameter change index *ρ*(*p*, *q*), This means that an improvement in the quality of the fit of equation (1) reducing the magnitude of the upper bound for *ρ*(*p*, *q*) will certainly increase the accuracy of the proxies *L*_*ga*_(*α*, *β*, *t*, ∆*t*). This study also demonstrates the importance of data quality control in the reduction of *ρ*_*max*_(*p*, *q*). Therefore, within the realm of parametric uncertainties as determined by of the values of $stde\left(\widehat{\alpha}\right)$ and $stde\left(\widehat{\beta}\right)$ obtained for the quality controlled data set, the addressed projection method can be considered robust relative to numerical differences in the estimators $\widehat{\alpha}$ and $\widehat{\beta}$.

**Fig. 10:**
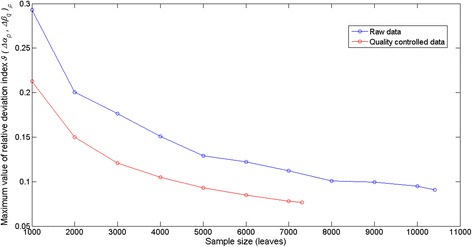
The effects of sample size on relative deviation index values. For a sample of a given size, the maximum value that the relative deviation index *ϑ*(*Δα*_*p*_, *Δβ*_*q*_ )_*ρ*_ attains is smaller for the quality controlled data. Even the smallest sized sample in the quality controlled data produces a higher precision that the largest sized one in the crude data

Figure 10 presents the variation of *ϑ*(*Δα*_*p*_, *Δβ*_*q*_) _*max*_ depending on sample size for both the set of raw data and that resulting after quality control procedures. As expected Fig. 10 shows that for both data sets *ϑ*(*Δα*_*p*_, *Δβ*_*q*_) _*max*_ decreases as sample size increases, but these plots also reveal that the effect of sample size on the accuracy of the *L*_*ga*_(*α*, *β*, *t*, Δ*t*) proxy is more pronounced for the raw data set. Moreover, even the smallest sized sample in the quality controlled data set (*n* = 1000) produces a smaller value for *ϑ*(*Δα*_*p*_, *Δβ*_*q*_)_*max*_ than that induced by the largest sized sample in the raw data set (*n* = 10412). This adds on to the aforesaid on the prominent role of data quality control in improving the accuracy of eelgrass leaf growth rate assessments obtained by means of *L*_*ga*_(*α*, *β*, *t*, ∆*t*).

## Conclusions

The results of the present study highlight the important role that the precision of parameter estimates linked to the allometric model of equation (1) plays in the overall suitability of the allometric proxy for eelgrass leaf growth rates *L*_*ga*_(*α*, *β*, *t*, ∆*t*) given by equation (17). The basic allometric model of equation (1) has been consistently identified using different data sets, and a property of invariance for the involved parameters statistically verified. But on spite of model consistency, the evaluation of the extent on what factors such as data quality sampling size, and analysis method can influence parameter estimates is an important entry in the comprehensive analysis of an allometric scaling relationship like equation (1). Moreover, the recommendation by Hui and Jackson [12] stating that for variables that are measured with errors, it is important to obtain accurate estimates with repeated measurements on similar individuals grown under similar conditions highlights the relevance of controlling factors that could affect data quality in our settings. Since the present results were obtained following the recommendation of Hui and Jackson [18], Packard and Birchard [19] and Packard and Boardman [20] concerning analysis method, and since sample size in our study was optimal, it is reasonable to assume that errors in data processing could explain a drop in the determination coefficient for the fitting of equation (1) to crude data collected at our study site from *R*^2^ = 92 obtained by Solana-Arellano et al. [11] and Echavarria-Heras et al. [15] to a value of *R*^2^ = 81 produced by the present data set. And the fact that after the data cleaning procedures performed on the present data we obtained for the resulting determination coefficient a value of *R*^2^ = 91 seems to endorse our judgment. Therefore, our results seem to confirm that data quality can be raised as a crucial factor explaining precision of estimates for the parameters *α* and *β* in equation (1). Moreover, the present data quality control procedure reveals the remarkable influence of inconsistencies in reducing precision of allometric projections. Indeed, as it is shown in Fig. 9 the accuracy of the *L*_*ga*_(*α*, *β*, *t*, ∆*t*) proxy improved after processing data irregularities. Our contribution provides guidelines that could prevent the proliferation of undesirable data quality related effects on the accuracy of allometric projections of eelgrass leaf growth rates. It was perhaps a lack of standardization that could explain a relatively reduced quality in the present data set. In other words the unduly deviations from an expected power function--like spreading could be probably tied to errors in leaf length estimation, faulty equipment for dry weight assessment, or even due to the incorrect recording of measurements. Nevertheless, the spread displayed in Fig. 4, shows notorious remnant variability for leaves longer than 500 mm, even after the application of data quality control procedures. This suggests that a data quality approach must be mandatory not only during the processing stage but even since the gathering step of forming a data set. Indeed for leaves with the smaller sizes, that normally yield reduced biomasses we might expect estimation errors that are imputable to the precision of the analytical scale used to obtain leaf dry weight estimations, along with the reading and/or recording of the output. But longest leaves remain exposed longer to environmental effects such as drag forces or herbivore. These influences could remove sections of the leaves rendering underestimated biomasses for a given leaf length. Therefore, special care must be also taken when processing longer an older leaves which are more often damaged of trimmed to such an extent that the biomass they report do not necessarily associate to the true length to weight relationship. We anticipate that the overlooking of these important issues as well as other data processing mistakes like rounding off errors or even incorrect data recording could explain the anomalous proliferation of inconsistencies (about 30 %) found in the present crude data set. Our findings also reveal that a data quality approach could optimize sampling endeavors, this because as shown in Fig. 10, even the smallest sized sample of the quality controlled data could entail better model identification results than the whole unprocessed data set. In summary, the present findings show how data quality control can be conceived as a fundamental step in the overall procedure that leads to reliable nondestructive allometric proxies of eelgrass leaf growth rates. And, it is also worth to point out, that it can be misleading to implicitly assume that a given set of data should conform to an idealized allometric relationship and then use a data cleaning procedure to ensure the fit. It can be misleading because the dispersion pattern itself could act as an indicator of eelgrass population status. But, we must emphasize that in accomplishing the present approach we are not neglecting the fact that there could be other unknown influences explaining the dispersion observed in the present crude data, but we are in fact acknowledging that the proven consistency and invariance of the addressed allometric relationship for eelgrass leaf biomass, is expected to provide a dependable account of pertinent causal influences, being these linked to the biology of the plant or associated to environmental forcing, thus highlighting the relevance of data quality influences in determining an unexpected variation pattern in the data as the one detected here.

## Figures and Tables

**Fig. 11:**
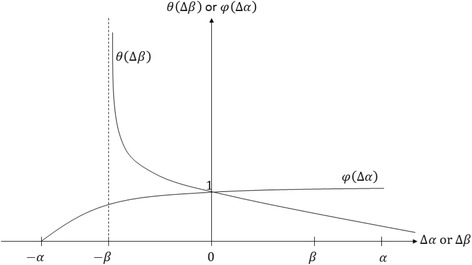
The behavior of the auxiliary factors *θ*(∆*β*) and *φ*(∆*α*). This plot presents the behavior of the auxiliary factors *θ*(∆*β*) and *φ*(∆*α*) defined by equations 24 and 25 respectively. Both ∆*α* and ∆*β* vary in the horizontal axis. Similarly the variations of *θ*(∆*β*) and *φ*(∆*α*) are projected in the vertical axis. We assumed that the order relationships 0 < *β* < *α*, and |∆β| < *β*, hold. Therefore, *θ*(∆*β*) remains positive and decreases and in turn we have *φ*(−*α*) = 0, and also that *φ(*∆*α*) remains positive and increasing for ∆*α* >−*α*. Particularly, for ∆*α* = ∆*β* = 0, we have *φ*(∆*α*) = *θ*(∆*β*) = 1

## Appendix A

In what follows a subscript *s* will be used to label a generic eelgrass shoot, holding a number *nl*(*s*) of leaves with combined biomass *w*_*s*_(*t*). In time, if ∆*w*_*l*_(t, ∆t) stand for the increment in biomass that is gained by an individual leaf over a growing interval [*t*, *t* + ∆*t*], then denoting by means of *L*_*sg*_ (*t*, ∆*t*) the resulting average growth rate of the leaves on the shoot *s* we will have,

$$ {L}_{sg}\left(t,\varDelta t\right)=\frac{{\displaystyle {\sum}_{nl(s)\ }}\varDelta {w}_l\left(t,\varDelta t\right)\ }{\varDelta t}, $$

and consequently if *L*_*g*_ (*t*, ∆*t*) denotes the linked average rate of leaf growth per shoot-day, we then have,

14 $$ {L}_g\left(t,\varDelta t\right)=\frac{{\displaystyle {\sum}_{NS\left(t,\varDelta t\right)}}{L}_{sg}\left(t,\varDelta t\right)}{NS\left(t,\varDelta t\right)}, $$

where ∑_*NS*(*t*,*Δt*)_ indicates summation of the shoots collected over the marking interval [*t*, *t* + ∆*t*] and being *NS*(*t*, ∆*t*) their number. Moreover, as it is thoroughly explained by Echavarría-Heras et al. [2], we can use equation (1) in order to derive an allometric approximation for *L*_*sg*_(*t*,∆*t*), which we here denote through the symbol *L*_*sga*_(*α*, *β*, *t*, *Δt*) and formally express by

15 $$ {L}_{sga}\left(\alpha, \beta, t,\varDelta t\right)=\frac{{\displaystyle {\sum}_{nl(s)}}\varDelta {w}_{la}\left(t,\varDelta t\right)}{\varDelta t}, $$

where *Δw*_*la*_(*t*, *Δt*) is an allometric proxy for *Δw*_*l*_(*t*, *Δt*).

It turns out that equation (1) yields,

$$ \varDelta {w}_{la}\left(t,\varDelta t\right) = \beta l{\left(t+\varDelta t\right)}^{\alpha } - \beta l{(t)}^{\alpha } $$

then, factoring *l*(*t* + Δ*t*)^α^ and taking into account that *l*(*t + Δt*) = *l*(*t*) *+ Δl* where *Δl* stands for the increment in leaf length gained over the interval [*t*, *t* + Δ*t*] we get,

$$ \varDelta {w}_{la}\left(t,\varDelta t\right) = \beta l{\left(t+\varDelta t\right)}^{\alpha}\left(1-{\left(1 - {\rho}_l\ \left(t,\varDelta t\right)\right)}^{\alpha}\right) $$

where

$$ {\rho}_l\ \left(t,\varDelta t\right)=\frac{\varDelta l}{l\left(t+\varDelta t\right)}. $$

And by letting

$$ \delta \left(t,\varDelta t\right)=\left(1-{\left(1-{\rho}_l\ \left(t,\varDelta t\right)\right)}^{\alpha}\right) $$

we equivalently have

$$ \varDelta {w}_{la}\left(t,\varDelta t\right) = \beta l{\left(t+\varDelta t\right)}^{\alpha}\delta \left(t,\varDelta t\right). $$

Therefore, from (15) we obtain,

16 $$ {L}_{sga}\left(\alpha, \beta, t,\varDelta t\right) = \frac{{\displaystyle {\sum}_{nl(s)}}\beta l{\left(t+\varDelta t\right)}^{\alpha}\delta \left(t,\varDelta t\right)}{\varDelta t} $$

Similarly, if *L*_*ga*_(*α*, *β*, *t*, *Δt*) denotes the allometric proxy for *L*_*ga*_(*t*, *Δt*), this is given by

17 $$ {L}_{ga}\left(\alpha, \beta, t,\varDelta t\right)=\frac{{\displaystyle {\sum}_{NS\left(t,\varDelta t\right)}}{L}_{sga}\left(\alpha, \beta, t,\varDelta t\right)\ }{NS\left(t,\varDelta t\right)}, $$

and in turn we have

18 $$ {L}_g\left(t,\varDelta t\right) = {L}_{ga}\left(\alpha, \beta, t,\varDelta t\right)+{\in}_{ga}, $$

with the term ϵ_*ga*_ standing for the involved approximation error. It is worth to point out that the derivation of the results of equation (18) could have been done using leaf area *a*(*t*) in place of *l*(*t*).

## Appendix B

In this appendix we firstly derive explicit formulae for the deviations δ*L*_*ga*_(∆*α*, ∆*β*, *t*, ∆*t*) that a change ∆*α* and ∆*β* in *α* and *β* will induce in *L*_*ga*_(*α*, *β*, *t*, ∆*t*). Using the derived forms we obtain domains of variation of ∆α and ∆β where *L*_*ga*_(α, β, *t*, ∆*t*) are underestimated or overestimated. Without loss of generality, we will assume through that the inequality 0 < *β* < *α* holds, as it regularly occurs in fittings of equation (1) to eelgrass leaf biomass and length data. Also, for the sake of mathematical tractability, without sacrificing pertinence of the analysis, regarding the increments ∆*α* and ∆*β* we will assume that |∆*α*| ≤ *α* and |∆*β*| < *β*.

By definition above we have,

19 $$ \delta {L}_{sga}\left(\varDelta \alpha,\ \varDelta \beta, t,\varDelta t\right)={L}_{sga}\left(\alpha +\varDelta \alpha,\ \beta +\varDelta \beta, t,\varDelta t\right)-{L}_{sga}\left(\varDelta \alpha,\ \varDelta \beta, t,\varDelta t\right) $$

And from equation (17) one gets

20 $$ \delta {L}_{ga}\left(\varDelta \alpha, \varDelta \beta, t,\varDelta t\right)=\frac{{\displaystyle {\sum}_{NS\left(t,\varDelta t\right)}}\delta {L}_{sga}\left(\varDelta \alpha,\ \varDelta \beta, t,\varDelta t\right)\ }{NS\left(t,\varDelta t\right)} $$

Now equation (16) yields,

21 $$ \delta {L}_{sga}\left(\varDelta \alpha,\ \varDelta \beta, t,\varDelta t\right)=\frac{{\displaystyle {\sum}_{nl(s)}}\beta l{\left(t+\varDelta t\right)}^{\alpha}\delta \left(t,\varDelta t\right){\mu}_{Lg}\left(\varDelta \alpha, \varDelta \beta, l\left(t+\varDelta t\right)\right)}{\varDelta t}, $$

With

22 $$ {\mu}_{Lg}\left(\varDelta \alpha, \varDelta \beta, l(t)\right)=\left(\left(\frac{\beta +\varDelta \beta }{\beta}\right)\left(\left(1-{\left(1-\frac{\varDelta l}{l\left(t+\varDelta t\right)}\right)}^{\alpha +\varDelta \alpha}\right)\ \right)\ \delta {\left(t,\varDelta t\right)}^{-1}l{\left(t+\varDelta t\right)}^{\varDelta \alpha } - 1\right), $$

which can be equivalently written as

23 $$ {\mu}_{Lg}\left(\varDelta \alpha, \varDelta \beta, l(t)\right)=\frac{\varphi \left(\varDelta \alpha \right)}{\theta \left(\varDelta \beta \right)}-1, $$

where

24 $$ \theta \left(\varDelta \beta \right)=\kern0.5em \frac{\beta }{\beta +\varDelta \beta }, $$

and

25 $$ \varphi \left(\varDelta \alpha \right)=\kern0.5em \frac{\left(1-{\left(\frac{l(t)}{l\left(t+\varDelta t\right)}\right)}^{\alpha +\varDelta \alpha}\right)l{\left(t+\varDelta t\right)}^{\varDelta \alpha }}{\left(1-{\left(\frac{l(t)}{l\left(t+\varDelta t\right)}\right)}^{\alpha}\right)}. $$

Now by assumption 0 < *β* < *α* and |∆*α* | ≤ *α*, and since observations show that the statement that *l*(*t*) being time increasing is consistent, for ∆*t* > 0 we necessarily have *l*(*t* + ∆*t*) > *l*(*t*) and therefore, the factor *φ*(∆*α*) as given above increases in its entire domain and also remains positive. Again the case ∆*α* = ∆*β* = 0 will set *θ*(∆*β*) = *φ*(∆*α*) = 1 (Fig. 11). Moreover, *L*_*ga*_(*α*, *β*, *t*, ∆*t*) will be overestimated by *L*_*ga*_(*α + ∆α*,*β + ∆β*,*t*) whenever the inequality

26 $$ {\mu}_{Lg}\left(\varDelta \alpha, \varDelta \beta, l(t)\right)>0 $$

holds, but since we also assumed that |Δ*β* | < *β*, as it has been already pointed out, the factor *θ*(Δ*β*) remains positive, by virtue of (23) we have that the statement in (26) implies

27 $$ \varphi \left(\varDelta \alpha \right) > \theta \left(\varDelta \beta \right). $$

Now for ∆*α* ≥ 0, *φ*(∆*α*) satisfies *φ*(∆*α*) ≥ 1 and increases, while for ∆*β* ≥ 0 the factor *θ*(∆*β*) satisfies *θ*(∆*β*) ≤ 1 and decreases, therefore the statement (27) will clearly hold in the domain ∆*α* > 0 and ∆*β* > 0. Notwithstanding, for ∆*β* = ∆*β*_*_ fixed, and such that 0 < ∆*β*_*_ < *β*, we have *θ*(*Δβ*_*_) ≥ 1/2, then since for -*α* ≤ ∆*α* < 0, we have 0 ≤ *φ*(*Δα*) ≤ 1, and *φ*(*Δα*) is continuous there, we can exhibit a value ∆*α*_*_, that satisfies, − *α* ≤ *Δα*_*_ < 0, for which we have *φ*(*Δα*_*_ ) = *θ*(*Δβ*_*_), and since *φ*(*Δα*) increases whenever ∆*α*_*_ ≤ ∆*α* ≤ 0, we will have *φ*(*Δα*) ≥ *θ*(*Δβ*_*_ ). Therefore, to each positive value of ∆*β*, we can associate a set of negative values of ∆α for which inequality (27) also holds.

Now, *L*_*ga*_ (*α* + ∆*α*, *β* + ∆*β*, *t*) will underestimate *L*_*ga*_(*α*, *β*, *t*, ∆*t*), whenever

28 $$ {\mu}_{Lg}\left(\varDelta \alpha, \varDelta \beta, l(t)\right)<0 $$

is satisfied, but again since |∆*β*| < *β*, from (23) the statement above implies

29 $$ \varphi \left(\varDelta \alpha \right)<\theta \left(\varDelta \beta \right) $$

Now for -*α* ≤ ∆*α* ≤ 0, the factor *φ*(∆*α*) vanishes at ∆*α* = −*α* and then increases monotonically towards a value *φ*(0) = 1. Meanwhile, as it has been discussed before for -*β* < ∆*β* ≤0 the term *θ*(∆*β*) decreases monotonically towards a value *θ*(0) = 1. Therefore, inequality (29) will particularly hold in the domain ∆*α* < 0 and ∆*β* < 0. This time, for ∆*β* = ∆*β*_*τ*_ fixed and satisfying -*β* < ∆*β*_*τ*_ <0, we have *θ*(Δ*β*_*τ*_) > 1 and since *φ*(∆*α*) is continuous, there exists ∆*α*_*τ*_ positive, for which we have *φ*(Δ*α*_*τ*_ ) = *θ*(Δ*β*_*τ*_) and since *φ*(∆*α*) increases and changes continuously over the domain |∆*α*| < *α*, particularly for 0 ≤ ∆*α* < ∆*α*_*τ*_ we will have, 1 ≤ *φ*(Δ*α*) < *θ*(Δ*β*_*τ*_). Therefore, each negative value of ∆*β* can be associated to a set of positive values of ∆*α* for which inequality (29) also holds.
